# Eating Behaviors and Physical Activity versus the Big Five Personality Traits in Women with a Hereditary Predisposition to Breast or Ovarian Cancer

**DOI:** 10.3390/nu16081244

**Published:** 2024-04-22

**Authors:** Beata Pięta, Agnieszka Bień, Michalina Pięta, Joanna Żurawska, Paweł Rzymski, Maciej Wilczak

**Affiliations:** 1 Department of Mother and Child Health, Poznan University of Medical Sciences, 33 Polna Street, 60-535 Poznan, Poland; bpieta@ump.edu.pl (B.P.); joannazurawska@ump.edu.pl (J.Ż.); parzymsk@gpsk.ump.edu.pl (P.R.); mwilczak@ump.edu.pl (M.W.); 2 Chair of Obstetrics Development, Faculty of Health Sciences, Medical University of Lublin, 4/6 Staszica Street, 20-081 Lublin, Poland; 3 University Clinical Hospital in Poznan, 49 Przybyszewskiego Street, 60-355 Poznan, Poland; michalinapieta@gmail.com

**Keywords:** eating behavior, physical activity, personality, *BRCA* 1 mutation, *BRCA 2* mutation

## Abstract

The Big Five personality traits—neuroticism, extroversion, openness to experience, agreeableness, and conscientiousness—represent continuous, individual features that affect a number of vital health aspects, including morbidity, self-reported health status, or lifestyle. The aim of this study was to analyze the relationship between the eating behaviors and engagement in physical activity of women with a hereditary predisposition to breast or ovarian cancer and the Big Five personality traits. A total of 357 women, participants of ‘The National Program for Families With Genetic/Familial High Risk for Cancer’, were included in the study. In the healthy group, the following statistically significant predictors were found in variables: agreeableness—meal frequency (β = 0.151; *p* = 0.030); neuroticism—consumption of fruits and vegetables (β = −0.177; *p* = 0.016) and cereal products (β = −0.223; *p* = 0.002); openness to experience—consumption of plant-based fats (β = 0.141; *p* = 0.034) and physical activity (β = 0.153; *p* = 0.021). In the cancer group, the frequency of dairy consumption (β = 0.286; *p* = 0.003) and physical activity (β = 0.370; *p* = 0.000) were found to be statistically significant predictors for the openness to experience variable. Neuroticism is associated with less frequent consumption of fresh fruits and vegetables as well as cereal products. Openness to experience was more often linked with a higher frequency of dairy consumption, plant-based fats, and physical activity. Women with breast or ovarian cancer and a higher openness to experience consumed dairy and engaged in physical activity more often than their peers with the remaining personality traits.

## 1. Introduction

Cancer continues to be one of the gravest health issues globally due to its high prevalence and mortality [1,2,3]. The nature of the disease is polyetiological, and one’s psychological condition may be perceived as one of many determinants of carcinogenesis. The correlations between malignancy and psychological factors such as personality traits, stress, depression, social isolation, and emotional reactions have been investigated for years by both researchers and practitioners. However, despite all efforts, the topic remains to be fully elucidated [4,5,6].

Certain personality traits have been known to improve wellbeing, while others may be conducive to disease emergence. The so-called ‘Big Five’ personality traits—neuroticism, extroversion, openness to experience, agreeableness, and conscientiousness—represent continuous, culturally universal, individual features of personality that affect crucial aspects of one’s health, including disease burden, perceived health, lifestyle and coping mechanisms, and reactions to stressful life events [5,7,8,9].

A personality type directly affects the way an individual copes with stressful situations and which stressors may be the most detrimental to their health [10]. Stress promotes the production of catecholamines such as epinephrine, noradrenaline, and dopamine, which—if chronic—may significantly impair immunologic functions, including the activity of the NK cells and lymphocyte proliferation [10,11]. In vitro, in vivo, and clinical studies have demonstrated that stress-related processes may also affect tumor progression signaling pathways, including immunoregulation, angiogenesis, and invasion. Therefore, individual response to chronic stress is directly linked to immunologic functions, and if the adaptive systems are not able to resolve the inflammation within the body, it may contribute to the pathogenesis of an oncologic disease [12,13]. According to the literature, approximately one-third of all malignancies develop due to high genetic predisposition. Various analyses of the factors responsible for genetic predisposition demonstrated a significant role of environmental factors, including personality traits [5,10].

A healthy lifestyle, defined as compliance with dietary recommendations and regular engagement in physical activity, indirectly lowers the risk of developing cancer [14]. An anti-cancer diet consists of supplying the body with nutrients that promote its normal and healthy functioning while avoiding products and behaviors that are detrimental or aggravating for the body. Such a diet is compliant with the guidelines of the Food Guide Pyramid, whose latest updates included physical activity in the recommendations [15,16]. Compliance with dietary recommendations helps to maintain good health or shorten the recuperation period, while poor eating habits may have long-term and progressing unfavorable health-related outcomes [17]. Proper diet remains one of the prerequisites for a healthy life for all human beings. In turn, a poorly balanced diet and poor eating habits, alcohol consumption, and infrequent physical activity may promote disease emergence. Studies about the relationship between stress and lifestyle in women with breast cancer have demonstrated that stress and a neurotic personality, combined with risky health-related lifestyle behaviors, might be contributing factors to developing breast cancer [10,11,18].

The relationship between physical activity and health as well as a decreased risk of malignant transformation in various types of cancer has been well-documented. Regular engagement in physical activity lowers the risk of developing breast, prostate, lung, and colorectal cancer as well as gastrointestinal malignant tumors. The relationship between lower risk of malignancy and higher physical activity is directly proportional. Regular physical activity may improve the survival rates in patients with breast and colorectal cancer [19]. Higher immune system function, the modulation of the endocrine system function—which is especially important in case of hormone-dependent tumors—and the modification of the body composition by reducing fatty tissue are among the most crucial effects of physical activity on oncologic patients [19,20,21].

### Purpose of the Study

The aim of this study was to analyze the relationship between eating behaviors and physical activity in women with a hereditary predisposition to developing breast or ovarian cancer and their psychological profile. Also, we aimed to identify those personality traits that are determinants of certain eating behaviors and engagement in physical activity.

## 2. Materials and Methods

### 2.1. Study Groups

The study was conducted among patients of the gynecological-obstetric hospital, Poznań University of Medical Sciences (PUMS), Poland, between 2013 and 2020. A total of 357 women, participants of ‘The National Program for Families With Genetic/Familial High Risk for Cancer’, were included in the study. The goal of the program is the early detection of malignant carcinoma in families with a hereditary risk of breast and ovarian cancer. Care for families with a high hereditary risk of developing breast and ovarian cancer is offered within the National Oncology Strategy Module I developed by the Ministry of Health in Poland. It involves the identification of individuals with high, hereditary genetic predisposition to those malignancies based on genetic testing, personal and familial medical history, and the subsequent monitoring of the high-risk individuals.

The following eligibility criteria for genetic testing for breast and/or ovarian cancer predisposition have been listed: ≥10% probability of pathogenic mutation; breast cancer diagnosis at ≤45; bilateral breast cancer at ≤45 or ovarian cancer at ≤50; known predisposing gene mutation for breast and/or ovarian cancer in the family; triple-negative breast cancer diagnosed at ≤60; non-mucinous epithelial cancer of the ovary or Fallopian tube or primary peritoneal cancer; ancestry linked with the ‘founder effect’, e.g., Ashkenazi Jewish; detection of the somatic *BRCA* mutations in each type of tumor with the allele frequency of >30% (if known); at least three breast cancer cases (with at least one diagnosed before the menopause) and/or ovarian cancer in close relatives; at least two first-degree relatives with any combination of the following high-risk features: bilateral breast cancer + another malignancy, breast cancer before age 50 + prostate cancer or pancreatic cancer before age 60; male breast cancer; breast and ovarian cancer in the same patient; two breast cancer cases diagnosed before age 50; eligible for testing for Li–Fraumeni syndrome or Cowden syndrome; and pancreatic cancer [22].

The respondents were recruited from The National Program for Families With Genetic/Familial High Risk for Cancer and followed up. The population was subdivided into two groups: the healthy group (*n* = 240)—women with elevated familial and/or genetic risk of developing breast/ovarian cancer, and the cancer group—women with a diagnosis of breast and/or ovarian malignancy confirmed in the last few months before the study (*n* = 117). Women with a familial history of three or more breast and/or ovarian cancer cases among first- and second-degree relatives and those with confirmed *BRCA1* and *BRCA2* or *PALB2* gene mutation (regardless of hereditary risk) were deemed eligible for the high-risk healthy group. Eligibility for the Program was the inclusion criterion for the study in both groups of women. The patients were followed up for the duration of the study.

### 2.2. Assessments

The study used a diagnostic survey with the following questionnaires:

– The Neo Five-Factor Inventory (NEO-FFI) includes 60 statements that are assessed on a five-point Likert scale, from 1—‘I completely disagree’ to 5—‘I completely agree’. The Inventory is based on the theory of the Big Five personality traits and allows us to evaluate the following dimensions of personality: openness to experience, conscientiousness, extroversion, agreeableness, and neuroticism. The scores of all entries were summed up to determine the total measure of all personality traits. The raw score of each scale had a possible range of 0–48 points. A higher score was indicative of a higher prevalence of a given personality trait. The α Cronbach coefficient for internal consistency was: 0.86 for neuroticism, 0.77 for extroversion, 0.73 for openness to experience, 0.68 for agreeableness, and 0.81 for conscientiousness [23].
– The questionnaire about eating behaviors and physical activity included questions about the frequency of consuming products that are perceived as ‘healthy foods’ according to the Food Pyramid: fresh fruits and vegetables, cereal products, dairy, white meat and fish, and plant-based fats. The weekly frequency of their consumption, classified on a scale from 0 to 5 (never, very seldom, sporadically, once a week, 2–3 times a week, 4–5 times a week, every day), and daily frequency of meal consumption (from 1 to 5) were investigated. Physical activity was investigated with the help of six questions about the frequency and types of physical activity. The respondents marked their answers on a six-point scale (with 0—‘never’ and 6—‘≥5 times/week’), with a maximum score of 36 points. The internal consistency measured by Cronbach’s alpha in the questionnaire was 0.74.
– A structured questionnaire that consisted of a set of standardized questions was used to characterize the study population.

Traditional paper questionnaires were used and completed by all respondents. All questionnaires were collected during the recruitment process. The study was approved by the PUMS Bioethics Committee (approval no. 706/16). The respondents were informed that participation was voluntary and that study results were anonymous and to be used exclusively for research purposes.

### 2.3. Statistical Analysis

Statistical analysis was conducted using StatSoftStatistica 13.1 PL. Descriptive analysis presented the data using mean, standard deviation, median, and minimum as well as maximum values. The Shapiro–Wilk test was used to investigate the distribution of the quantitative data. After determining the distribution—non-normal—the Mann–Whitney U test (Z) was used to compare both groups. The Student’s (*t*) test was used to compare variables with little statistical significance between the groups, while one-way analysis of variance (ANOVA) (F) without correction was used to test the dependence of one or more variables (equal variances). Linear regression analysis was used to test the model and a mediator model. The *p*-value of <0.05 was considered statistically significant.

## 3. Results

The mean age in the healthy group was 47.99 years, compared to 53.29 years in the cancer group (*p* < 0.001). No statistically significant differences between the groups were found in terms of education (*p* = 0.58) and place of residence (*p* = 0.79). The mean age at menarche in the healthy group was 13.38 years (SD = 1.49), as compared to 13.36 years in the cancer group (SD = 1.67) (*p* = 0.396). In the cancer group, the majority of patients had breast cancer—68.4%, 28.2% had ovarian cancer, and 3.4% of the respondents had both malignancies.

The mean values for the investigated personality traits in both groups of women are presented in Table 1. In both groups, conscientiousness (healthy group M = 35.21 vs. cancer 34.76) was the most prevalent and neuroticism (healthy group M = 21.97 vs. cancer 21.19) was the least prevalent personality trait. No statistically significant differences between personality-trait prevalence in the healthy and cancer group were found (*p* > 0.05).

In the cancer group, higher meal frequency per day (*p* < 0.004) and more frequent consumption of fresh fruits and vegetables (*p* < 0.036) were observed, as compared to the healthy group. No statistically significant differences between the study and healthy group were found in the following: frequency of consumption of cereal products, dairy, white meat and fish, animal-based fats, and physical activity (*p* > 0.05) (Table 2).

Regression analysis of the personality traits (NEO-FFI) among the healthy group is presented in Table 3. The number of meals per day was a statistically significant predictor for agreeableness (β = 0.151; *p* = 0.030). More-agreeable individuals consumed a higher number of meals per day. The frequency of consumption of fresh fruits and vegetables (β = −0.177; *p* = 0.016) and cereal products (β = −0.223; *p* = 0.002) was a statistically significant predictor for neuroticism. Higher neuroticism was associated with a lower frequency of consumption of fresh fruits and vegetables as well as cereal products. Regression analysis demonstrated that the frequency of dairy (β = 0.164; *p* = 0.014) and plant-based fat (β = 0.141; *p* = 0.034) consumption and engagement in physical activity (β = 0.153; *p* = 0.021) were independent predictors for openness to experience. Individuals with higher openness to experience scores more often consumed dairy products and plant-based fats and engaged in physical activity.

The regression analysis of the personality traits (NEO-FFI) among cancer patients is presented in Table 4. The frequency of dairy consumption (β = 0.286; *p* = 0.003) and engagement in physical activity (β = 0.370; *p* = 0.000) were statistically significant predictors of openness to experience. Women with higher openness to experience scores more often consumed dairy and engaged in physical activity.

## 4. Discussion

Most malignant neoplasms have a complex etiology that includes genetic, environmental, and lifestyle factors and their interactions. The recently observed increased focus on lifestyle changes to improve these factors is the consequence of a growing recognition of the role of the host in cancer survival [24]. In our study, no statistically significant differences in the prevalence of the investigated personality traits between the healthy group and the cancer group were found. This suggests that the oncologic disease or its absence is not directly related to differences in the personality trait scores.

A diet that is both well-balanced and rich in nutrients may significantly lower the risk of developing a malignancy or promote better therapy outcomes. Dietary intervention in patients with neoplasms may become an integral part of cancer therapy [25,26]. According to the European Society for Clinical Nutrition and Metabolism’s evidence-based guidelines for clinical nutrition in oncologic patients, nutrition counseling has been awarded a grade ‘A’ for the quality of evidence and strength of recommendations [27].

In our study, cancer patients consumed a higher number of meals and were more likely to choose fresh fruits and vegetables as compared to the healthy group. One of the possible explanations might be a higher health awareness of the affected individuals after receiving the cancer diagnosis. The women might have been more willing to change their eating habits, in compliance with the dietary recommendations, in an effort to improve their health. At diagnosis, most oncologic patients strive to lead a healthier lifestyle, which might include a more balanced diet. Moreover, a higher number of meals and more frequent consumption of fresh fruits and vegetables by cancer patients might be caused by higher health awareness and the desire to increase the supply of vital nutrients to help the body fight with the disease [26]. This demonstrates compliance with the guideline to include low-GI (glycemic index) products in the diet, as they will not contribute to weight gain and remain within the recommended dietary pattern [28]. According to the findings of a meta-analysis by Shin et al., the consumption of fresh fruits and vegetables was associated with a 29% lower risk of developing breast cancer [29]. Likewise, Farvid et al., demonstrated that a healthy lifestyle associated with the frequent consumption of fruits and vegetables was correlated with a 38% lower risk of developing breast cancer, while failure to stay compliant with the dietary recommendations resulted in a 44% higher risk of malignant transformation [30]. Fruits and vegetables contain nutrients and bioactive agents that demonstrate anticancer activities. Moreover, these food groups share properties that may promote the formation of an environment that prevents cancer growth and progression. The highest anticarcinogenic potential has been reported for plant-derived foods, e.g., plant-derived polyphenols, glucosinolates, carotenoids, folates, selenium, chlorophyll, dietary fiber, anthocyanins, and phytoestrogens, as well as vitamins with antioxidant properties [30].

The Big Five Personality Trait model is one of the most popular concepts that view personality as the result of internal characteristics. The complexity of human behavior, including the interactions between personality features and lifestyle choices, has long been a matter of interest for various authors [8,31]. There were no significant differences in personality-trait prevalence between the healthy group and the cancer group. The lack of bias (completely healthy versus cancer) between the groups makes further research feasible. Statistically significant correlations between personality traits and eating behaviors have been demonstrated. These features have been linked with dietary choices, eating disorders, attitudes to genetically modified foods, and preferences concerning the consumption of local and ecological food. Openness to experience and agreeableness have been linked with relatively healthier dietary patterns and more-balanced dietary choices, while extraversion and neuroticism were linked with less-healthy dietary patterns [32,33,34]. To the best of our knowledge, only a few studies so far have investigated the link between the Big Five Personality Traits model and the frequency of food consumption. Conscientiousness and openness to experience were positively correlated with the number of daily servings of fruits and vegetables. The same two features were positively linked with the use of the so-called Mediterranean diet, and conscientiousness was also positively correlated with a lower consumption of meat, e.g., pork [8].

Agreeableness encompasses features such as kindness, cooperation, and altruism. That personality trait may also incorporate eating habits as people with high-level agreeableness may be more likely to take into consideration the preferences of others when making decisions about meal preparation and choice of foods [31,35]. In our study, healthy patients with high scores for agreeableness were also more likely to comply with the recommendations about the regularity of meals. Keller and Siegrist, whose study aimed to analyze the direct and indirect effects of personality on eating habits and dietary choices, demonstrated a direct link between agreeableness and meat consumption. Higher agreeableness proved to be adversely correlated with the frequency of meat consumption [8]. In turn, Cousin et al., and Bahat emphasized that patients with high agreeableness communicated better with their physicians, formed positive interactions with others, and complied with recommendations as compared to individuals with low levels of agreeableness [36,37].

Neuroticism is defined as the predisposition to experience strong negative emotions and self-pity manifested as high anxiety, increased levels of guilt, irritability, timidity, and inability to cope with stress [5,31]. Neuroticism may impede self-control and promote the consumption of calorie-dense, fatty, and high-sugar products to deal with negative emotions and stress [8]. In a study by Otonari et al., women with higher neuroticism scores were at an elevated risk of developing cancer, whereas neuroticism was positively correlated with the risk factors for malignancy. Women with higher neuroticism scores consumed fewer fruits and vegetables, were less likely to engage in physical activity, smoked cigarettes, and had higher BMIs [38]. A similar correlation was found in our study in the healthy group at risk of malignant transformation. Higher neuroticism was demonstrated to be linked with a lower frequency of consumption of fresh fruits and vegetables as well as cereal products. Importantly, highly neurotic individuals may demonstrate an aversion to certain foods due to their enhanced sensitivity to the taste and consistency of foods, which limits their food choices. On the other hand, they may be more likely to consume comfort foods, looking for ways to alleviate emotional stress [8]. Studies have also demonstrated that neurotic and emotionally non-stable individuals report a low regularity of meals, and their poor eating behaviors may affect their dietary choices. Also, neuroticism was found to be linked with elevated levels of biomarkers for chronic inflammation such as interleukin-6 and C-reactive protein [39].

Individuals with high openness to experience scores are creative, flexible, appealing, and progressive. Openness to experience frequently manifests as an appreciation of diversity and intellectual curiosity. Such people are more likely to follow diverse and unconventional diets, encompassing a broad range of products [23,31]. In our study, the eating habits of the healthy group with higher openness to experience were more compliant with the food pyramid, with dairy and plant-based fats at the base of the pyramid, meaning such individuals are more concerned with proper nutrition. As for cancer patients, respondents with higher openness to experience scores reported a higher frequency of dairy consumption. The inclusion of various dairy products such as low-fat milk, yogurt, and cheese—which are sources of valuable nutrients like protein, calcium, vitamin D, probiotics, and iodine—in a well-balanced diet may decrease the risk of developing some types of cancer [40,41]. We hypothesize that our cancer group could have been better self-educated and made attempts at healthier nutrition, regardless of the fact that such behaviors do not act as prophylaxis at that stage of the disease. Guyonnet et al. reported that an increased intake of vitamin D and calcium lowered the risk of developing breast cancer, mainly among the carriers of the *BRCA1* gene [42]. It has also been demonstrated that higher consumption of essential amino acids, which are mainly found in dairy products—leucine, isoleucine, and valine—is associated with longer survival among breast cancer patients. Additionally, a high supply of these products helps to maintain adequate weight, especially if combined with physical activity [43,44,45]. Plant-based fats are believed to be healthier because they contain unsaturated fatty acids, vitamins, and antioxidants, which promote overall health and wellbeing and reduce the risk of developing neoplasms. Pastore et al. found that frequent consumption of grains, vegetables, fruits, and plant-based oils and low consumption of saturated and trans fats, red meat, processed foods, and alcohol was inversely proportional to mammographic breast density, which is a known risk marker for breast cancer [46,47].

In the healthy group, individuals with higher agreeableness consumed more meals per day; those with higher neuroticism had lower consumption of fruits and vegetables and cereal; and those with higher openness to experience consumed more dairy and plant-based fats and engaged in physical activity. In the cancer group, those with higher openness to experience consumed more dairy and engaged in physical activity. In summary, the construct of personality traits that originates from the Big Five Model plays a diverse role in shaping individual eating behaviors. It may also be a useful resource when attempting to describe and explain health behaviors among women with an elevated risk of developing malignancy or with an already diagnosed malignancy. Understanding how individual differences affect dietary preferences and habits as well as awareness of potential challenges allows for a more personalized and effective approach to nutrition. Still, it is important to bear in mind that malignant transformation should be considered in the broad context of the environment and life situations of every individual. The higher level of stress and pressure associated with these situations is correlated with a lower immune response in the body, which further increases the risk of malignancy [24].

In recent years, we have witnessed an increasing number of reports about the significance of physical activity in cancer prophylaxis, as well as during and after cancer therapy. Physical activity has been proven to lower the risk of developing colorectal, breast, renal, endometrial, vesical, and stomach cancers as well as esophageal adenocarcinoma [48,49]. Contrary to common misconceptions and frequent concerns of afflicted patients, specialists emphasize that physical activity is safe for cancer patients. Obviously, a complex work-up of the patient’s condition, with emphasis on detecting the existing limitations and possible contraindications, is necessary before engaging in physical activity [48,49,50].

In our study, the mean weekly engagement in physical activity in both groups of women was 11 points (M = 11.68 healthy group vs. M = 10.79 cancer patients), with a maximum score of 36. This score is decidedly below the levels of physical activity recommended by the current guidelines concerning physical activity in cancer prophylaxis. The current guidelines suggest 150 min of moderate-intensity training or 75 min of high-intensity training per week. Cancer patients are advised to engage in moderate-intensity training for a minimum of 30 min at least five times a week. These patients should comply with the so-called pyramid of physical activity, with non-exercise activity thermogenesis (NEAT) activities at its base, i.e., energy expenditure during spontaneous everyday physical activities [51].

In order to promote an active lifestyle, it is essential to first recognize the factors linked with physical activity or its absence. Personality, which might be the key factor linked with willingness to engage in physical activity and help to identify individuals at risk of physical activity avoidance, has been suggested as one of the psychological factors that determine individual differences in thinking and reasoning, and predisposition to certain behaviors [52,53]. According to the findings of our study, women with higher openness to experience scores—both those with elevated familial and/or genetic risk of developing breast and/or ovarian cancer and cancer patients—were more likely to engage in physical activity as compared to their peers with other personality traits. Possibly, individuals with a dominant trait of openness to experience are more likely to explore new life experiences and demonstrate positive appraisal, cognitive curiosity, and higher novelty tolerance. Individuals with high openness to experience exhibit a tendency for pro-health and prophylaxis attitudes, as well as compliance with the guidelines due to their innate desire to seek out new information [37,54]. In our study, we found relatively low levels of engagement in physical activity among our respondents, which indicates that educational efforts about the benefits of physical education at all stages of cancer therapy and prevention are necessary. The identification of the links between the Big Five personality traits and physical activity might supply valuable information about individual preferences and motivation to engage in physical activity. The recognition of the impact of personality on the approach to physical activity may play a role in developing personalized strategies for promoting more-active and healthy lifestyles.

The identification of personality types and modification of those types as well as pro-health patterns of behavior are possible to a certain extent but remain challenging. Mentality, mindset, and behaviors may be modified, including the ability to express emotions and manage stress. Deficits in that area promote the emergence of numerous diseases, and their modification may play an important role in the process of prophylaxis, e.g., lifestyle modifications [8,9,31]. In the case of patients with a diagnosis, psychological interventions targeted at personality traits may improve the effectiveness of therapy and rehabilitation, lower the risk of complications and disease recurrence, and consequently improve the quality of patient life. Stanisz et al. demonstrated that carriers of the *BRCA1* and *BRCA2* mutations with high neuroticism scores had decreased quality of life (QoL), especially in the domains of low mood, anxiety, and sleep disorders. At the same time, higher scores in conscientiousness, openness to experience, extroversion, and agreeableness were associated with improved QoL [55]. Notably, personality traits may also affect self-reported perception of eating behaviors and physical activity [5,56]. Higher awareness of proper nutrition in cancer patients and adequate-intensity physical activity may improve their eating habits and response to therapy, prevent the progression of malignancy-related emaciation, and consequently promote a better quality of patient life [57,58].

Clinical data about the effectiveness of psychosocial interventions to decelerate cancer progression remain inconclusive, and the behavioral mechanisms behind them need to be elucidated. The identification of these interconnections might facilitate individualized interventions, which would take into account both psychological and physical aspects of patient disposition. As cancer therapy evolves towards a more individualized approach, it is vital to identify those patients who would benefit most from behavioral and/or pharmacological interventions that block the unfavorable effect of psychosocial factors on therapy outcomes [12]. In the case of cancer patients, psychological intervention focused on personality traits may increase the effectiveness of the treatment and rehabilitation and decrease the risk of complications and disease recurrence.

### Strengths and Limitations of Our Study

To the best of our knowledge, this is the first study to investigate the eating habits and engagement in physical activity in women with a hereditary predisposition to breast and/or ovarian malignancy and cancer patients versus the personality traits of the Big Five model, which is believed to be one of the most renown personality concepts.

Our study is not without limitations. The study was conducted in only one province in Poland, so the study sample will not be representative of the population of women. A homogenous population was yet another limitation as it hinders result generalizability. Also, we did not investigate at which stage of treatment the respondents were deemed eligible for the study. It was a cross-sectional study, which reduces the probability of obtaining conclusive findings and limits the understanding of predictors influencing eating habits and physical activity in women with a hereditary predisposition to breast and/or ovarian cancer. The retrospective nature of the questions in the group of women with cancer is associated with the risk of recall bias.

## 5. Conclusions

No differences in neuroticism, extroversion, openness to experience, conscientiousness, and agreeableness between women at elevated risk of breast or ovarian cancer and women with a diagnosis of breast or ovarian cancer were found. Agreeableness in high-risk breast/ovarian cancer women correlated with a higher number of meals per day. Women with high neuroticism scores were less likely to consume fresh fruits and vegetables or cereal products. Openness to new experiences was more often related to the consumption of dairy, the use of plant-based fats while preparing meals, and higher physical activity.

Women with a diagnosis of breast or ovarian cancer and higher openness to experience scores more often consumed dairy and engaged in physical activity as compared to their peers with the remaining personality traits.

## Figures and Tables

**Table 1:** Prevalence of the Big Five personality traits in the study population.

NEO-FFI	Group	M	SD	Me	Min	Max	t	*p*
Neuroticism	healthy group	21.97	22.00	7.69	6.00	47.00	−0.903	0.367
	cancer group	21.19	21.00	7.81	2.00	45.00		
Extraversion	healthy group	28.88	29.00	5.72	12.00	43.00	−0.630	0.529
	cancer group	28.47	29.00	6.01	15.00	46.00		
Openness to Experience	healthy group	25.64	25.00	5.05	12.00	39.00	−0.865	0.388
	cancer group	25.14	25.00	5.30	12.00	41.00		
Agreeableness	healthy group	31.93	32.00	4.91	10.00	44.00	−0.792	0.429
	cancer group	31.49	31.00	5.03	14.00	45.00		
Conscientiousness	healthy group	35.21	36.00	5.16	20.00	48.00	−0.764	0.445
	cancer group	34.76	34.00	5.26	22.00	48.00		

M—mean; SD—standard deviation; Me—median.

**Table 2:** Frequency of healthy food consumption and engagement in physical activity in the study population.

Variable	Healthy Group			Cancer Group			Statistical Analysis	
	M	SD	Me	M	SD	Me	Z	*p*
Meals (number/day)	3.93	4.00	0.98	4.23	4.00	0.81	−2.852	0.004
Fresh fruits and vegetables (amount/week)	4.55	5.00	0.81	4.72	5.00	0.73	−2.101	0.036
Cereal products (amount/week)	3.57	4.00	1.56	3.73	4.00	1.53	0.958	0.338
Dairy (amount/week)	1.90	2.00	1.07	1.98	2.00	1.19	−0.530	0.596
White meat and fish (amount/week)	4.02	4.00	0.65	4.01	4.00	0.78	−0.460	0.646
Plant-based fats (amount/week)	2.56	3.00	1.02	2.36	2.00	1.09	1.583	0.113
Physical activity	11.68	11.00	7.77	10.79	9.00	7.49	−1.043	0.297

M—mean; SD—standard deviation; Me—median.

**Table 3:** Univariate regression of personality traits (NEO-FFI), eating habits and physical activity in healthy women—healthy group.

**Personality Traits**	**Meal Frequency** ***F* = 1.740; *p* < 0.10; *R* = 0.189; *R*^2^ = 0.036**				
	***B***	***SE***	***β***	***t***	***p***
(constant)	2.997	0.800		3.747	0.000
Neuroticism	0.004	0.009	0.031	0.420	0.675
Extraversion	−0.001	0.012	−0.005	−0.067	0.946
Openness to Experience	0.022	0.013	0.111	1.681	0.094
Agreeableness	0.030	0.014	0.151	2.179	0.030
Conscientiousness	−0.018	0.013	−0.097	−1.410	0.160
**Personality Traits**	**Frequency of Consumption of Fresh Fruits and Vegetables** ***F* = 2.496; *p* < 0.01; *R* = 0.225; *R*^2^ = 0.051**				
	***B***	***SE***	***β***	***t***	***p***
(constant)	5.134	0.657		7.815	0.000
Neuroticism	−0.019	0.008	−0.177	−2.436	0.016
Extraversion	0.008	0.010	0.055	0.781	0.436
Openness to Experience	0.008	0.011	0.049	0.746	0.456
Agreeableness	0.002	0.011	0.014	0.196	0.845
Conscientiousness	−0.019	0.011	−0.121	−1.780	0.076
**Personality Traits**	**Frequency of Consumption of Cereal Products** ***F* = 3.999; *p* < 0.001; *R* = 0.281; *R*^2^ = 0.079**				
	***B***	***SE***	***β***	***t***	***p***
(constant)	3.515	1.243		2.827	0.005
Neuroticism	−0.045	0.015	−0.223	−3.114	0.002
Extraversion	−0.001	0.019	−0.005	−0.065	0.948
Openness to Experience	0.034	0.020	0.109	1.691	0.092
Agreeableness	0.020	0.022	0.063	0.931	0.353
Conscientiousness	−0.012	0.020	−0.040	−0.589	0.556
**Personality Traits**	**Frequency of Dairy Consumption** ***F* = 1.417; *p* < 0.10; *R* = 0.171; *R*^2^ = 0.029**				
	***B***	***SE***	***β***	***t***	***p***
(constant)	1.184	0.877		1.351	0.178
Neuroticism	0.006	0.010	0.041	0.556	0.578
Extraversion	−0.015	0.013	−0.078	−1.093	0.276
Openness to Experience	0.035	0.014	0.164	2.476	0.014
Agreeableness	−0.002	0.015	−0.008	−0.108	0.914
Conscientiousness	0.005	0.014	0.023	0.338	0.736
**Personality Traits**	**Frequency of White Meat and Fish Consumption** ***F* = 2.190; *p* < 0.01; *R* = 0.211; *R*^2^ = 0.045**				
	***B***	***SE***	***β***	***t***	***p***
(constant)	3.080	0.530		5.809	0.000
Neuroticism	0.005	0.006	0.062	0.850	0.396
Extraversion	0.015	0.008	0.135	1.904	0.058
Openness to Experience	0.013	0.008	0.098	1.496	0.136
Agreeableness	0.016	0.009	0.122	1.766	0.079
Conscientiousness	−0.013	0.009	−0.105	−1.542	0.125
**Personality Traits**	**Frequency of Plant-Based Fat Consumption** ***F* = 1.377; *p* < 0.10; *R* = 0.169; *R*^2^ = 0.029**				
	***B***	***SE***	***β***	***t***	***p***
(constant)	1.522	0.833		1.828	0.069
Neuroticism	0.001	0.010	0.006	0.076	0.940
Extraversion	−0.015	0.013	−0.086	−1.205	0.229
Openness to Experience	0.028	0.013	0.141	2.128	0.034
Agreeableness	0.005	0.014	0.026	0.372	0.710
Conscientiousness	0.016	0.014	0.081	1.180	0.239
**Personality Traits**	**Physical Activity** ***F* = 1.725; *p* < 0.10; *R* = 0.189; *R*^2^ = 0.036**				
	***B***	***SE***	***β***	***t***	***p***
(constant)	2.493	6.339		0.393	0.695
Neuroticism	−0.029	0.074	−0.029	−0.395	0.693
Extraversion	−0.006	0.097	−0.004	−0.060	0.952
Openness to Experience	0.235	0.101	0.153	2.320	0.021
Agreeableness	−0.034	0.110	−0.022	−0.314	0.754
Conscientiousness	0.144	0.103	0.096	1.394	0.165

**Table 4:** Univariate regression of personality traits (NEO-FFI), eating habits and physical activity in the cancer group.

**Personality Traits**	**Meal Frequency** ***F* = 0.590; *p* < 0.10; R = 0.161; *R*^2^ = 0.025**				
	***B***	***SE***	***β***	***t***	***p***
(constant)	3.837	1.081		3.550	0.001
Neuroticism	0.010	0.013	0.094	0.779	0.438
Extraversion	−0.011	0.015	−0.080	−0.732	0.466
Openness to Experience	0.019	0.015	0.125	1.245	0.216
Agreeableness	−0.001	0.017	−0.008	−0.076	0.940
Conscientiousness	0.002	0.017	0.010	0.089	0.929
**Personality Traits**	**Frequency of Consumption of Fresh Fruits and Vegetables** ***F* = 1.085; *p* < 0.10; *R* = 0.216; *R*^2^ = 0.047**				
	***B***	***SE***	***β***	***t***	***p***
(constant)	4.670	0.959		4.871	0.000
Neuroticism	−0.007	0.011	−0.071	−0.594	0.554
Extraversion	−0.006	0.013	−0.046	−0.424	0.673
Openness to Experience	−0.011	0.014	−0.081	−0.813	0.418
Agreeableness	−0.009	0.015	−0.059	−0.560	0.577
Conscientiousness	0.026	0.015	0.186	1.717	0.089
**Personality Traits**	**Frequency of Consumption of Cereal Products** ***F* = 0.388; *p* < 0.10; *R* = 0.131; *R*^2^ = 0.017**				
	***B***	***SE***	***β***	***t***	***p***
(constant)	1.987	2.048		0.970	0.334
Neuroticism	0.001	0.024	0.003	0.027	0.979
Extraversion	0.003	0.028	0.011	0.099	0.922
Openness to Experience	0.016	0.029	0.055	0.543	0.588
Agreeableness	0.003	0.033	0.009	0.083	0.934
Conscientiousness	0.033	0.032	0.114	1.038	0.301
**Personality Traits**	**Frequency of Dairy Consumption** ***F* = 3.909; *p* < 0.001; *R* = 0.387; *R*^2^ = 0.150**				
	***B***	***SE***	***β***	***t***	***p***
(constant)	−0.976	1.476		−0.662	0.510
Neuroticism	0.000	0.017	0.002	0.018	0.986
Extraversion	0.014	0.020	0.073	0.712	0.478
Openness to Experience	0.064	0.021	0.286	3.041	0.003
Agreeableness	0.046	0.024	0.196	1.967	0.052
Conscientiousness	−0.015	0.023	−0.068	−0.662	0.509
**Personality Traits**	**Frequency of White Meat and Fish Consumption** ***F* = 1.510; *p* < 0.10; *R* = 0.1252; *R*^2^ = 0.064**				
	***B***	***SE***	***β***	***t***	***p***
(constant)	5.530	1.019		5.427	0.000
Neuroticism	−0.012	0.012	−0.118	−0.993	0.323
Extraversion	−0.026	0.014	−0.200	−1.862	0.065
Openness to Experience	−0.019	0.015	−0.127	−1.287	0.201
Agreeableness	0.008	0.016	0.055	0.521	0.604
Conscientiousness	−0.009	0.016	−0.063	−0.588	0.558
**Personality Traits**	**Frequency of Plant-Based Fat Consumption** ***F* = 0.544; *p* < 0.10; *R* = 0.155; *R*^2^ = 0.024**				
	***B***	***SE***	***β***	***t***	***p***
(constant)	0.764	1.456		0.525	0.600
Neuroticism	0.007	0.017	0.052	0.428	0.670
Extraversion	0.011	0.020	0.060	0.544	0.588
Openness to Experience	0.013	0.021	0.064	0.634	0.528
Agreeableness	0.030	0.023	0.137	1.280	0.203
Conscientiousness	−0.004	0.023	−0.019	−0.175	0.862
**Personality Traits**	**Physical Activity** ***F* = 5.228; *p* < 0.001; *R* = 0.437; *R*^2^ = 0.191**				
	***B***	***SE***	***β***	***t***	***p***
(constant)	−17.341	9.073		−1.911	0.059
Neuroticism	0.045	0.106	0.047	0.423	0.673
Extraversion	0.081	0.124	0.065	0.648	0.518
Openness to Experience	0.523	0.130	0.370	4.035	0.000
Agreeableness	0.210	0.145	0.141	1.443	0.152
Conscientiousness	0.147	0.142	0.104	1.035	0.303

## Data Availability

The original contributions presented in the study are included in the article, further inquiries can be directed to the corresponding author.
